# Possible Zoonotic Transmission of Hepatitis E from Pet Pig to Its Owner

**DOI:** 10.3201/eid1307.070063

**Published:** 2007-07

**Authors:** Christophe Renou, Jean-Francois Cadranel, Marc Bourlière, Philippe Halfon, Denis Ouzan, Hervé Rifflet, Philippe Carenco, Abdelouahid Harafa, Jean Jacques Bertrand, Annie Boutrouille, Pierre Muller, Jean-Pierre Igual, Anne Decoppet, Marc Eloit, Nicole Pavio

**Affiliations:** * Centre Hospitalier d’Hyères, Hyères, France; † Centre Hospitalier, Creil, France; ‡ Hôpital Saint-Joseph, Marseille, France; § Hôpital Ambroise Paré, Marseille, France; ¶ Institut Arnaud Tzanck, Saint-Laurent du Var, France; # Centre Hospitalier, Ajaccio, Corsica, France; ** Agence Française de sécurité Sanitaire des Aliments, Ecole Nationale Vétérinaire d'Alfort, Institut National de la recherche Agronomique, Maisons-Alfort, France; †† Direction Départementale des Affaires Sanitaires et Sociales du Var, Toulon, France

**Keywords:** Hepatitis E virus, zoonosis, transmission pathway, dispatch

## Abstract

Hepatitis E is transmitted mainly by water or food, but in industrialized countries, all routes of transmission have not been identified. We describe possible zoonotic transmission of hepatitis E virus that involved direct contact between a pet pig and its owner.

Hepatitis E virus (HEV) infections occur sporadically in industrialized countries, where this virus is not endemic, although these infections were initially reported only in persons who had traveled to countries where the virus is endemic. An increasing number of autochthonous cases of HEV have been recently reported in industrialized countries such as Japan, Greece, Spain, Italy, the United States, and France (*1*).

Several lines of evidence suggest that swine, which are also sensitive to HEV infection, may act as a reservoir of the virus and that HEV infections in humans in industrialized countries might be zoonotic. Antibodies to HEV have been detected in domestic pigs in regions where HEV is not endemic (*2*). These antibodies have been detected more frequently in humans occupationally exposed to swine than in those not exposed to swine (*3*). Human and swine HEV strains are closely related genetically (*4*–*7*), and experimental infections of swine with strains from humans and nonhuman primates have been reported (*8*). Moreover, sporadic human cases of acute HEV infection linked with consumption of raw or insufficiently cooked meat (boar or deer) have been reported in Japan (*9*). Therefore, HEV is likely a zoonotic virus that can be directly transmitted by some types of meat or possibly by direct contact with infected animals.

As in other industrialized countries, sporadic autochthonous HEV infections have recently occurred in France (*1*,*10*). However, the routes of transmission for such infections have not been clearly identified, and this information is needed to characterize the clinical epidemiology of HEV.

## The Patient

We report a 41-year-old patient in France with isolated episodes of ≈1-month duration of fatigue since the end of September 2005. Analysis of a serum sample obtained from the patient on day 1 of consultation (October 21, 2005) showed markedly increased liver enzyme levels (aspartate aminotransferase 393 IU/L, alanine aminotranferase 1,211 IU/L), although no associated cholestatic biochemical symptoms were present. The patient lived alone in an urban area, had not traveled abroad for at least 1 year, drank alcoholic beverages only occasionally, and had not recently received any intravenous injections or taken any drugs. Test results were negative for serologic markers of hepatitis A, B, and C, and tests for Epstein-Barr virus and cytomegalovirus detected immunoglobulin G (IgG) for previous infections. Antibodies to HEV were detected in his serum sample by using an enzyme immunoassay (HEV ELISA; Genelabs Diagnostics, St Ingbert, Germany). A positive result (optical density/cutoff value >1) was obtained for HEV-specific IgM (absorbance 8.9); HEV-specific IgG was not detected (optical density/cutoff value <1).

**Figure 1:**
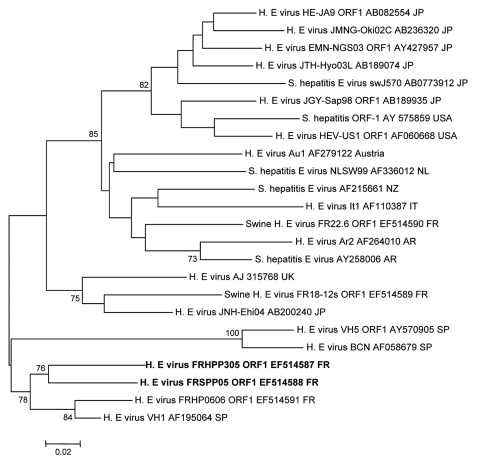
Phylogenetic tree (neighbor-joining method) of hepatitis E virus (HEV) genotype 3 isolates for a 210-nt sequence within the open reading frame (ORF) 1 gene (corresponding to nt 167–376 of the prototype swine genotype 3 pSHEV-3 AY575859). Patient (EF514587) and pet pig (EF514588) sequences are in **boldface** and were compared with French isolates or known isolates from regions where HEV is not endemic. GenBank accession no. and country of origin are indicated. Reliability of the different phylogenetic groupings was evaluated by using a bootstrap test (1,000 replications); scores >70% are indicated. Scale bar indicates no. of nucleotide substitutions per site. JP, Japan; USA, United States; NL, the Netherlands; NZ, New Zealand; IT, Italy; FR, France; AR, Argentina; UK, United Kingdom; SP, Spain.

**Figure 2:**
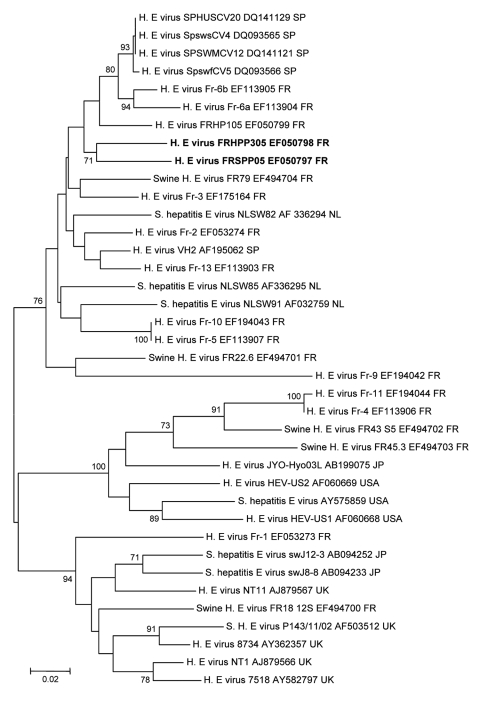
Phylogenetic tree (neighbor-joining method) of hepatitis E virus (HEV) genotype 3 isolates for a 273-nt sequence within the open reading frame 2 gene (corresponding to nt 6078–6350 of the prototype swine genotype 3 pSHEV-3 AY 575859). Patient (EF050798) and pet pig (EF050797) sequences are in **boldface** and were compared with French isolates or known isolates from regions where HEV is not endemic. GenBank accession no. and country of origin are indicated. Reliability of the different phylogenetic grouping was evaluated by using a bootstrap test (1,000 replications); scores >70% are indicated. Scale bar indicates no. of nucleotide substitutions per site. SP, Spain; FR, France; NL, the Netherlands; JP, Japan; USA, United States; UK, United Kingdom.

HEV RNA was detected in the serum sample by using a nested reverse transcription–PCR for open reading frame (ORF) 1 or ORF2 genes (*11*,*12*). The amplified fragments were sequenced directly and aligned with those of other HEV strains. A genotype 3 isolate was identified by using MEGA 3.1 software (www.megasoftware.net). This genotype was similar to those of other European isolates but was specific to this case, which suggested autochthonous local transmission (GenBank accession nos. EF514587 and EF050798) (Figures 1, 2).

Detection of HEV-specific IgM and viral RNA in the patient resulted in a diagnosis of hepatitis E. Gradual clinical and biochemical improvement occurred spontaneously in this patient. Eight weeks before the onset of fatigue, the patient had been given a 3-month-old Vietnamese pig that had been born in France. The pig urinated and defecated outside, and the patient regularly changed the litter. The animal often entered the house and was frequently handled by its owner. In early November 2005, serum samples were collected from the pig and tested for HEV. HEV-specific IgG was not detected, but HEV RNA was detected. As with the patient, a genotype 3 strain of HEV was identified in serum from the pig by phylogenetic analysis (GenBank accession nos. EF514588 and EF050797) (Figures 1, 2). Isolates obtained from the patient and the pet pig showed homology of 92% at the nucleotide level and 98% at the amino acid level in the ORF2 gene, which has a similar degree of variability as that of the entire HEV genome.

Phylogenetic analyses of HEV genotype 3 sequences for isolates from France and other countries where the virus is not endemic showed a similarity to sequences for the ORF1 and ORF2 genes in isolates from the French patient and his pig. These patient and pet sequences were different from sequences for isolates from patients in the same area of France (EF514591-ORF1, EF050799-ORF2) and from contemporary sequences detected in isolates from French swine herds (EF514589-, EF514590-ORF1; EF494700-, EF494701-, EF494702-, EF494703, EF494704-ORF2) (Figures 1, 2).

## Conclusions

The divergence (8%) observed between sequences from the patient and his pet pig is consistent with a variable degree of homology (90%–94%) within 10 HEV subtypes of genotype 3. Furthermore, similar to other RNA viruses, such as hepatitis A and C, HEV is a quasispecies (*13*). In cases of acute hepatitis E contracted by consumption of infected meat, 100% homology between isolates from humans and infected animals was observed, which indicates that during ingestion, all quasispecies are transmitted. In the present case, the route of transmission was different and might have involved selection of variants with a greater zoonotic infection potential by the fecal-oral route. We did not investigate quasispecies variability in this study because of low residual viremia (not detected by single PCR). The unusually prolonged viremia in the pet pig might have resulted in accumulation of mutations over time.

Evidence suggests that direct zoonotic transmission was involved in this case. The possible incubation time of 8 weeks corresponds to the time between acquisition of the pig and onset of the symptoms. Excretion of HEV by the pet pig probably occurred soon after the patient received it, when the animal was ≈3 months of age. This is consistent with what is observed in natural infections, in which high viral titers are observed at ≈12–15 weeks of age. Although viremia usually lasts >3 weeks in a swine experimental model of HEV (*14*), persistence of residual viremia might be explained by the lack of seroconversion observed and the absence of HEV-specific IgG in the pig. Consistent with the genetic variability of HEV and quasispecies, sequences amplified from virus isolates from the patient and the pig were phylogenetically related (ORF1 and ORF2 genes). The difference observed between the 2 sequences suggests that accumulation of mutations occurred during the unusual prolonged viremia observed in the pet pig. There was frequent contact between the pet pig and its owner, including changing the litter, and such contact is presumably responsible for animal-to-human transmission in persons with occupational exposure to swine (*3*). Other possible sources of infection were unlikely. These sources include contaminated water (the pig owner used only the local urban water supply and had no domestic wells) and consumption of undercooked or raw pork (the patient reported that he did not eat pork).

Isolation of virus with related HEV sequences from the patient and his pet pig suggests that the most likely route of transmission was from pig to human. This case therefore supports the current assumption that HEV may be a zoonotic virus and that domestic pet swine are 1 of the natural hosts of HEV. However, the source of HEV infection for such animals is not known and still being investigated.
